# Case Report: Malignant Brain Tumors in Siblings With *MSH6* Mutations

**DOI:** 10.3389/fonc.2022.920305

**Published:** 2022-07-12

**Authors:** Di Wu, Qingshan Chen, Jian Chen

**Affiliations:** ^1^ Institute of Functional Nano and Soft Materials (FUNSOM) and Collaborative Innovation Center of Suzhou Nano Science and Technology, Soochow University, Suzhou, China; ^2^ Department of Neurosurgery, The Second People’s Hospital of Liaocheng of Shandong Province, Liaocheng, China; ^3^ Chinese Institute for Brain Research, Beijing, Research Unit of Medical Neurobiology, Chinese Academy of Medical Sciences, Beijing, China

**Keywords:** brain tumor, genome sequencing, *MSH6*, DNA Mismatch Repair, CMMRD, case report

## Abstract

**Background:**

Familial brain tumor incidences are low. Identifying the genetic alterations of familial brain tumors can help better understand the pathogenesis and make therapy regimens for these tumors.

**Case Presentation:**

An elder female and a younger male were diagnosed with brain tumors at the age of 10 and 5, respectively. Whole-genome sequencing analysis of the two patients’ blood, primary brain tumor tissues, and their parents’ blood samples was performed, which revealed that the two tumor samples harbored extremely high somatic mutation loads. Additionally, we observed pigmentation on the male patient’s skin.

**Conclusion:**

Germline, biallelic mutation of *MSH6*—a gene related to DNA mismatch repair whose defect will result in constitutional mismatch repair deficiency (CMMRD)—is causal for the brain tumors of these two siblings.

## Introduction

Brain tumors are the most common and lethal type of solid tumors in children (1). They range from the least common, non-invasive, surgically curable pilocytic astrocytoma to the common, highly malignant glioblastoma (GBM) and medulloblastoma (MB) (2–5). Both of GBM and MB are classified as grade IV in malignancy by the World Health Organization (WHO) (6). Familial brain tumor incidences, on the other hand, are relatively low. Gorlin syndrome patients caused by inherited *PTCH1* mutations can develop MB and Li-Fraumeni syndrome patients resulted from germline *TP53* inactivation are associated with malignant gliomas (7, 8). Constitutional mismatch repair deficiency (CMMRD) syndrome, which was called Turcot syndrome for many years, is also associated with an increased risk of brain cancer (9).

CMMRD syndrome is a distinct childhood cancer predisposition syndrome characterized by diverse malignancies in hematological organs, the brain, the large intestine and other organs (10). Patients mostly fail in reaching their adulthood (11). The most prevalent are brain tumors and the age at diagnosis has been estimated to be 10.3 years old (12). The majority of the brain tumors are malignant gliomas, but MB and other central nervous system tumors have also been reported (10, 13). GBM is the most lethal tumor in CMMRD patients (14). The disease is caused by biallelic germline mutations that occur in one of the four mismatch repair (MMR) genes (*MLH1*, *MSH2*, *MSH6*, *PMS2*) (15). The protein products of these MMR genes are highly conserved from bacteria to humans, which are responsible for the correction of mismatches, insertions and deletions during DNA replication and recombination (16). Humans have two types of MMR enzymes: MutS (hMSH2, hMSH3 and hMSH6) and MutL (hMLH1, hMLH3, PMS1 and PMS2) (17). MutS enzymes first recognize mismatched nucleotides in DNA and then work in combination with MutL enzymes to activate other proteins to remove the mismatched DNA strand and synthesize a new DNA strand (18, 19). In MutS, hMSH6 and hMSH2 function as a heterodimer to recognize single base mismatches as well as 1-2 base insertions and deletions, while the complex of hMSH3 and hMSH2 recognizes larger insertion or deletion loops up to 13 nucleotides (16, 20). Patients with *MSH6* nullizygous mutations commonly develop brain tumors before the age of 10 (9).

The diagnosis of CMMRD syndrome is difficult due to many reasons. Firstly, CMMRD syndrome is caused by biallelic germline mutations of MMR genes but their parents with only one allelic mutation show a low risk of cancer predisposition (21). Secondly, CMMRD syndrome lacks unique clinical features and clear diagnostic criteria. Its clinical presentation varies and the phenotypes also overlap with other tumor syndromes such as Li-Fraumeni syndrome (22).

In this case report, an elder sister and a younger brother were diagnosed with GBM and MB, respectively. Further genomic sequencing confirmed that both patients harbored biallelic *MSH6* mutations, thus confirming the diagnosis of CMRRD syndrome.

## Case Presentation

**Figure 1:**
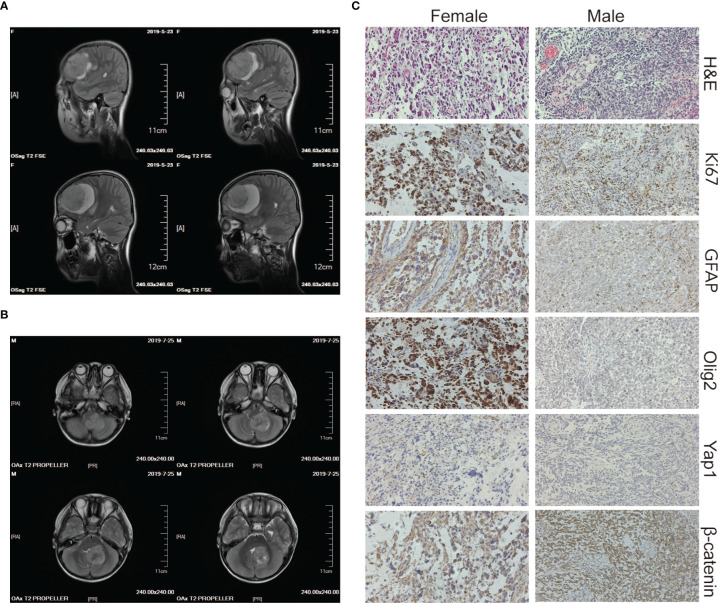
Imaging and histology of the brain tumors. **(A, B)** CT scan images of the brain tumor tissue of the female (upper panels) and the male (lower panels). **(C)** H&E, Ki67, GFAP, Olig2, Yap1, and β-catenin staining of the female’s (left panels) and the male’s (right panels) brain tumor tissue. The magnification is 20×.

The two patients were siblings, and their parents were nonconsanguineous, healthy, and had no family history of genetic or infectious diseases. The elder female and the younger male were diagnosed with brain tumors at the age of 10 and 5 years old, respectively. The male patient repeatedly vomited without any obvious causes five days before being admitted to the hospital and became slightly worse mentally and ate less after the illness. The female patient had similar symptoms with paroxysmal headaches. Computed tomography (CT) showed that the female had a tumor in the right frontal lobe and the male had a tumor in the cerebellar region (**Figures 1A, B**), and subsequent histopathology confirmed the diagnoses of GBM and MB, respectively (**Figures 1C**). Then both of them underwent surgery for total tumor resection. One month later, Intensity-modulated radiation therapy (IMRT) was followed in the female, where a DT dose of 50 Gy in 2 Gy daily fractions in GTV and 45 Gy in 1.8 Gy daily fractions in PTV were delivered with instruction from the radiation oncologist. The frequency of administration is 5 times weekly. For the male, the radiotherapy was administered in the dose of 36 Gy in 1.8 Gy daily fractions. The siblings tolerated radiation therapy very well and the follow-up brain MRI revealed no brain tumor. However, one and a half years later, tumor recurrence was suspected in the female through MRI and she received the second radiotherapy (dose of DT: 39.6 Gy in 1.8 Gy daily fractions) combined with temozolomide (TMZ) chemotherapy (100 mg daily for 5 days). For the male, a neoplasm lesion was detected in the spinal canal after 6 months. The second surgery was performed and followed by radiotherapy one month later (dose of DT: 42 Gy in 2.1 Gy daily fractions). The siblings have already resumed normal schooling and daily activities. The timeline from the episode of care in the two cases have been illustrated in **Supplementary Figure 1**.

**Figure 2:**
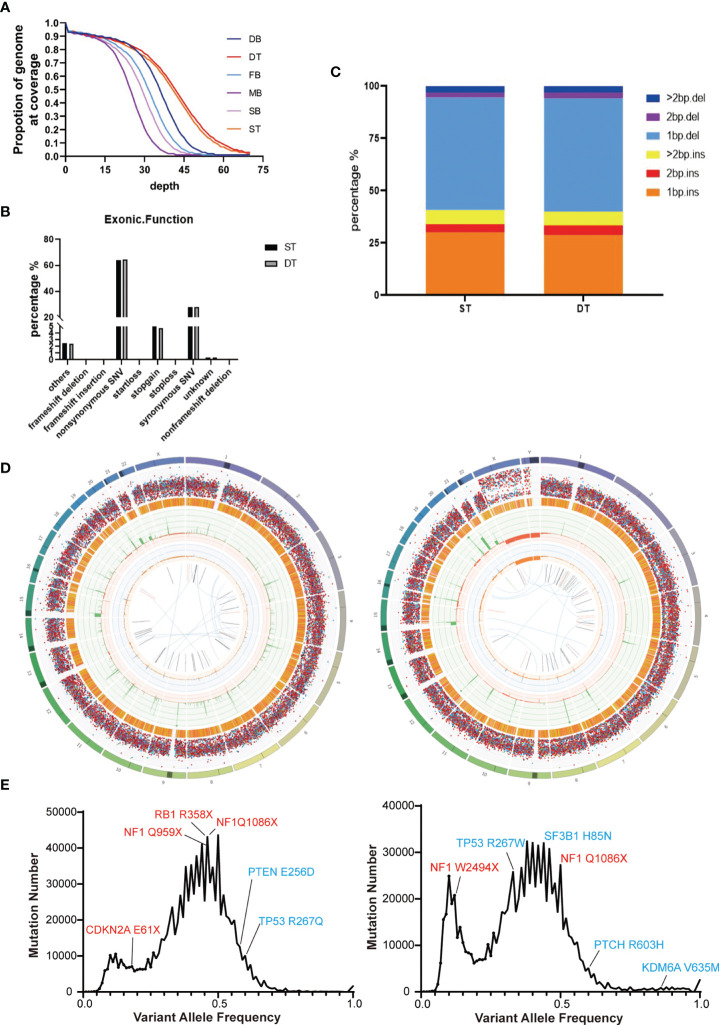
Summary of the whole-genome sequencing results of the two brain tumor cases. **(A)** Sequencing depth and coverage of the tumor and blood samples. D refers to the female patient (daughter); S refers to the male patient (son); F refers to the patients’ father; M refers to the patients’ mother. B refers to blood samples. T refers to tumor tissue. **(B)** The proportion of different somatic mutation types in exons. **(C)** The distribution of somatic Indel length. **(D)** Circos plots of the female’s (left) and the male’s (right) brain tumor tissue illustrated distributions of all exonic mutations across the chromosomes. The outer first circle showed the chromosomes and the darker shaded areas represented large gaps. The second circle showed the somatic variants and each dot represented a single somatic variant. The third circle showed all observed tumor purity adjusted copy number changes, including both focal and chromosomal somatic events. The fourth circle represented the observed minor allele copy numbers across the chromosome. The innermost circle displayed the observed structural variants within the chromosomes. **(E)** Mutation number vs. Variant allele frequency (VAF) plot of the female’s (left) and the male’s (right) brain tumor tissue. Potential driver mutations are labeled (Red: Truncation mutation; Blue: Non-synonymous mutation).

We performed whole-genome sequencing of the patients’ tumor tissue and blood samples, as well as their parents’ blood samples, to obtain an overview of the somatic mutation landscape. The tumors were sequenced at 50X and the blood samples were sequenced at 30X (**Figure 2A**). Both tumor cases harbored millions of somatic mutations, many hundreds of folds higher than the average somatic mutation numbers in either GBM or MB (23, 24) (**Figure 2D**). In both tumors, the predominant mutations were single base mutations (**Figure 2C**) and about half of the mutations in exons were non-synonymous mutations (**Figure 2B**). We also observed some well-known somatic mutations such as mutations in *NF1*, *RB1*, *CDKN2A*, *TP53* and *PTEN* in the GBM case and mutations in *TP53*, *NF1*, *SF3B1* and *PTCH* in the MB case (**Figure 2E**).

**Figure 3:**
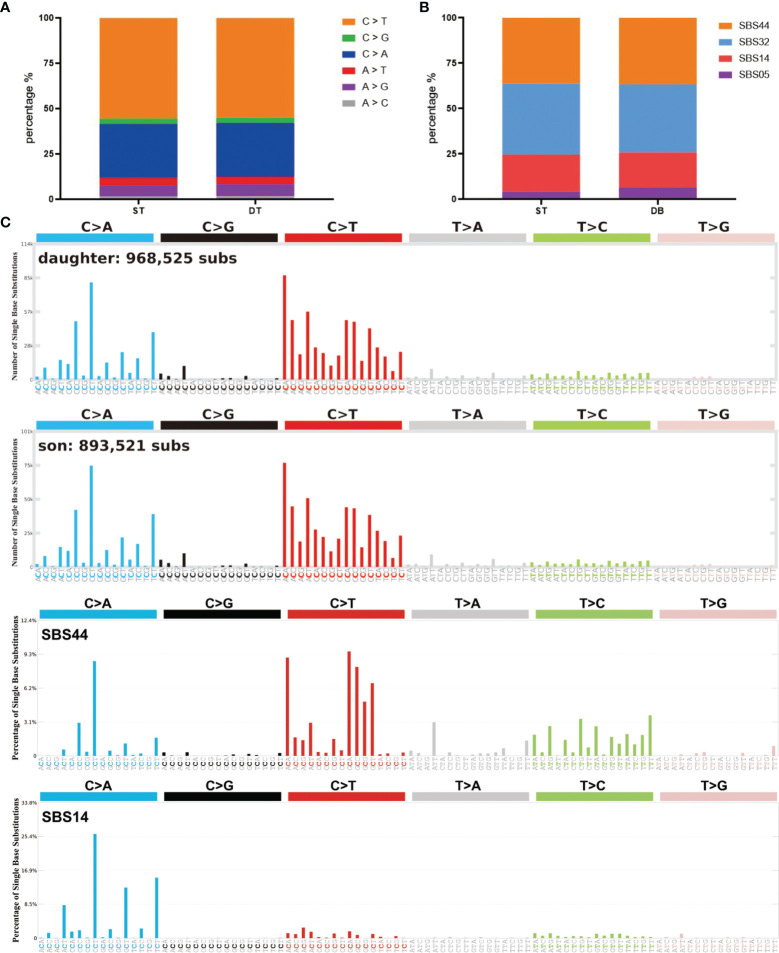
Summary of SBS signatures of the two brain tumor cases. **(A)** The distribution of different single base substitution types. **(B, C)** SBS signatures of the two brain tumor cases and two comparable mutational signatures: SBS44 and SBS14.

Somatic mutations can occur in all cells of the body throughout the whole lifetime. They may arise due to mistakes in DNA replication, modification or repair processes. The development of cancer is often accompanied by somatic mutations (25). Based on this, the concept of Mutational Signatures has been proposed, which represents the unique set of characteristics of mutational profiles on the genome (25). Single base substitution (SBS) signatures have been identified by frequencies of 96 different contexts, considering the mutated base and the bases immediately 5’ and 3’ (26, 27). Based on the assessment of 1,865 whole genomes and 19,184 exomes, 60 SBS signatures have been defined (available at the Cosmic website) (26). We compared the mutational signatures of the two cases with the known mutational signatures and the result indicated strong signals of the SBS44 and SBS14 signatures for both cases; note that both of these signatures are associated with DNA mismatch repair (**Figures 3A–C**).

**Figure 4:**
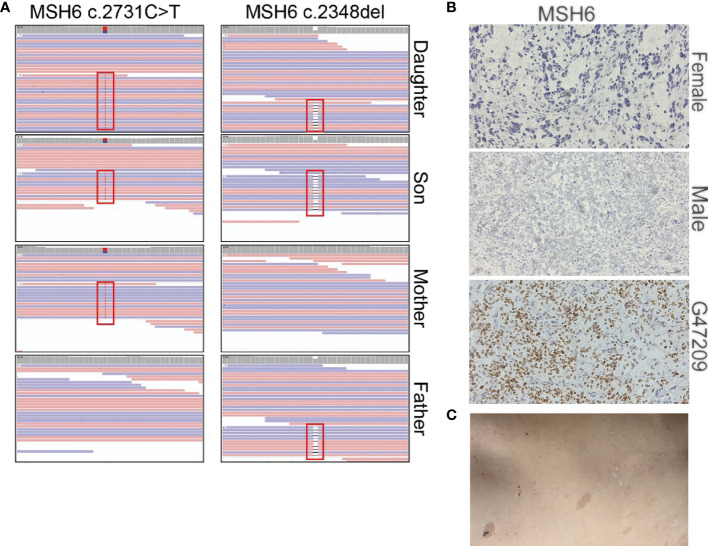
Summary of the *MSH6* mutation in the two brain tumor cases. **(A)** Mutation positions of *MSH6* in the two brain tumor cases and their parents. **(B)** MSH6 staining in the two brain tumor cases and the positive control from a glioma case (G47209) expressing MSH6. The magnification is 20×. **(C)** Hyperpigmented skin lesions in the male case.

Our sequencing results showed that the parents had a heterozygous defect and the two patients had homozygous defects in *MSH6*. The mother carried an *MSH6 c.2731C>T* nonsense mutation and the father carried a single nucleotide deletion that resulted in a frameshift of the protein (**Figure 4A**). We performed immunohistochemical (IHC) staining to verify the loss of MSH6 protein in both tumor cases (**Figure 4B**). *MSH6* nullizygous usually causes CMMRD syndrome, which is typically accompanied by a visible symptom called café-au-lait macules (CALM) (22). We detected multiple flat patches of skin that were darker than the surrounding area in the male (**Figure 4C**).

## Conclusion and Discussion

CMMRD syndrome is a rare childhood cancer susceptibility syndrome. The lack of awareness and broad cancer spectrum of malignancies contributes to diagnosis difficulty. Most CMMRD patients have multiple CALM reminiscent of neurofibromatosis type 1 (NF1) (10, 28). It has been demonstrated that *NF1* is a frequent somatic mutation target of MMR deficiency. Parents of CMMRD patients commonly have no symptoms of NF1, while the offspring may present NF1-associated signs when they inherit both of the mutant MMR alleles from their parents (28). In the two cases, for clinicians, CMMRD syndrome was not taken into consideration, and the two patients were diagnosed as common brain tumors in the beginning because of scarce knowledge of this syndrome and little attention to the diffuse and irregular hyperpigmented macules and the absence of a family history of neoplasms. The diagnosis was confirmed only when germline biallelic inactivation of *MSH6* and a huge somatic mutation load in the tumor were discovered by high throughput sequencing. Of note, microsatellite instability (MSI) is a recognized biomarker for MMR deficiency, which is also can be an auxiliary index for diagnoses (29). In addition, immunohistochemistry to detect the expression of MMR proteins could be an inexpensive alternative method to help CMMRD diagnosis.

The parents of the two cases had no history of colorectal cancers but they did carry a heterozygous mutation of *MSH6*. Compared with *MSH2* and *MLH1*, the clinical severity of heterozygous *MSH6* and *PMS2* mutations is lower, and the diagnosis of CMMRD syndrome often lacks a family history of cancer (21). It is significant for clinicians to be aware of CMMRD syndrome and assess cancer risk in these patients and their relatives. Early cancer surveillance and timely interventions may benefit their future lifetime. There have been clinical diagnostic criteria and guidelines for surveillance proposed by the European Consortium “Care for CMMR-D” (C4CMMR-D) (10, 14). Commencing MRI scanning at 2 years old and scanning once every 6–12 months is suggested, but whether it will help improve survival has not been validated (14).

In the two cases, radiotherapy received an effective therapeutic outcome, but the information available for optimal treatment is still an urgent requirement. Radiotherapy and adjuvant TMZ chemotherapy are the commonly used treatment for brain tumors (30). However, for CMMRD patients with brain tumors, chemotherapy is typically not a feasible choice because commonly used chemotherapeutic alkylating agents can only initiate efficient tumor damage with a functional MMR system (31). According to the statistics collected by the European C4CMMRD Consortium, five out of six patients showed poor response to chemotherapy (14). The therapeutic efficiency of TMZ has also been reported to be limited in two patients with *MSH6*-mutated recurrent GBM, and its use should be avoided due to its known ability to accumulate somatic mutations and promote neoplastic progression (32, 33). In the previous case reports regarding children with brain tumors carrying biallelic *MSH6* mutations, the survival was mostly 12-36 months after surgery and subsequent chemoradiotherapy, and many demonstrated resistance to TMZ (11, 32–40). Effective chemotherapeutic drugs for CMMRD syndrome are still lacking, but early detection of tumors may allow for the most effective chemotherapeutic approach (33).

In summary, accurate diagnoses, long-time surveillance and effective therapies for CMMRD patients are still difficulties to be overcome. Several case reports have elaborated immune checkpoint inhibitor (ICPI) can improve the survival of CMMRD patients with malignant gliomas (41–44). One of them reported that a 5-year-old female GBM patient with biallelic *MSH6* mutations was treated with nivolumab and showed a durable response to ICPI treatment and regression of the tumor. For the siblings in this report, ICPI may be taken into consideration if the tumor reoccurs.

## Methods

### Whole-Genome Sequencing (WGS)

Genomic DNA was extracted from the tumor and blood samples using a genomic DNA extraction kit (Tiangen Biotech, DP304). The library was constructed and sequenced using Illumina NovaSeq 6000 platform with 150 bp pair-end reads (GeneWiz Inc.).

### Bioinformatic Analysis

The raw 150 bp pair-end reads were trimmed using fastp (45) and aligned to hg38 human genome using Sentieon’s bwa mem algorithm (Sentieon Inc, San Jose, CA). The germline variations were called following Sentieon’s DNAseq pipeline (https://support.sentieon.com/manual/DNAseq_usage/dnaseq/) and the somatic variations were called using TNseq pipeline (https://support.sentieon.com/manual/TNseq_usage/tnseq/). The germline and somatic variations were annotated by Annovar (46). The genomic alignment result was visualized by Integrative Genomics viewer (IGV) (47). MutSignatures (48) was used to analyze and visualize mutation signatures. Circos map was produced using Circos (49).

## Data Availability Statement

The datasets presented in this study can be found online at: https://ngdc.cncb.ac.cn/gsa-human/. The accession number is HRA002310.

## Ethics Statement

Patient information was de-identified. The studies involving human participants were reviewed and approved by the institutional review board of The Second People’s Hospital of Liaocheng of Shandong Province. Written informed consent to participate in this study was provided by the participants' legal guardian.

## Author Contributions

JC conceived and designed the experiments. DW performed the experiments and analyzed the data. QSC collected the clinical samples and analyzed clinical data. DW and JC wrote the paper. All authors read and approved the final manuscript.

## Funding

This work was supported by the National Key R&D Program of China (2016YFA0503100) and the Major Project for Natural Science Research in Jiangsu Province (17KJA320004).

## Conflict of Interest

The authors declare that the research was conducted in the absence of any commercial or financial relationships that could be construed as a potential conflict of interest.

## Publisher’s Note

All claims expressed in this article are solely those of the authors and do not necessarily represent those of their affiliated organizations, or those of the publisher, the editors and the reviewers. Any product that may be evaluated in this article, or claim that may be made by its manufacturer, is not guaranteed or endorsed by the publisher.
