# Supplementary figures and images for: Case Report: Malignant Brain Tumors in Siblings With MSH6 Mutations
Source: Front Oncol. 2022 Jul 12;12:920305. doi: 10.3389/fonc.2022.920305 (PMC9315106; doi:10.3389/fonc.2022.920305)

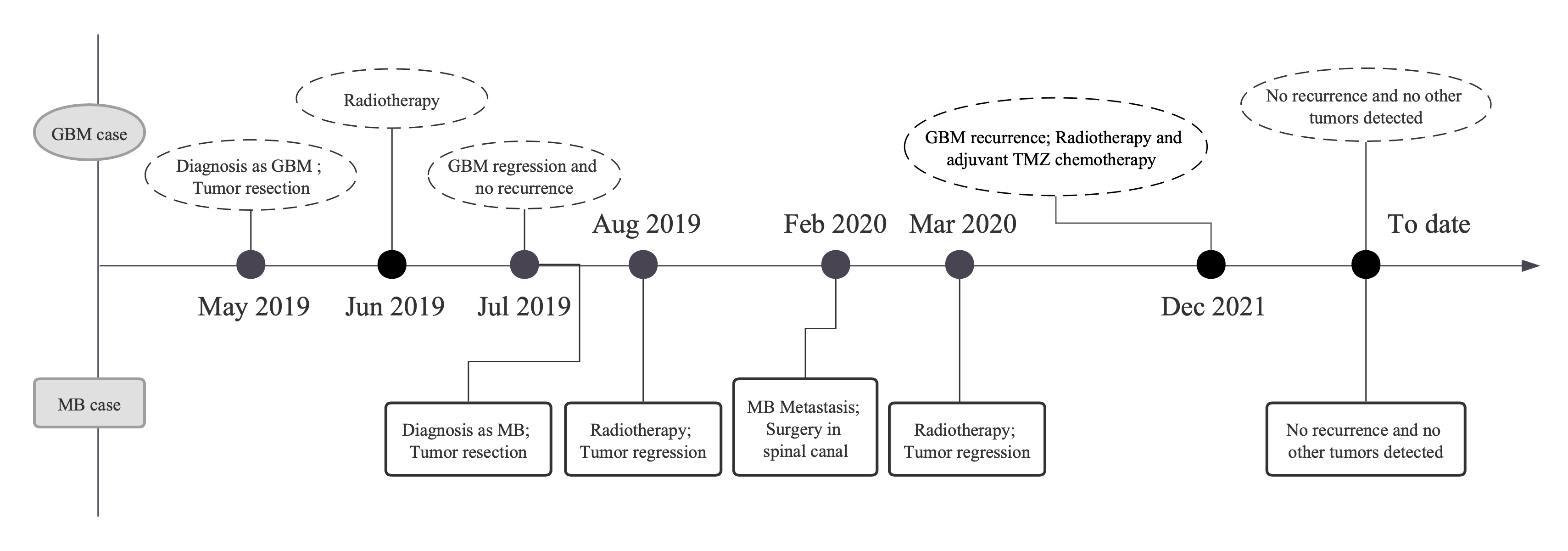

Supplement: Supplementary file 1 [file Image_1.tif]
